# Thermodynamic and Ab Initio Design of Multicomponent Alloys Based on (Fe_50_Mn_30_Co_10_Cr_10_)-xBx (x = 0, 5, 7, 10, and 15 at.%)

**DOI:** 10.3390/ma16165579

**Published:** 2023-08-11

**Authors:** Rodrigo Vargas-Osorio, Laura Gabriela Torres-Mejia, Lais Mujica-Roncery, Jose Y. Aguilar-Hurtado, Katherine Paredes-Gil

**Affiliations:** 1 Departamento de Química, Facultad de Ciencias Naturales, Matemática y del Medio Ambiente, Universidad Tecnológica Metropolitana, Santiago 7800003, Chile; 2 Grupo de Investigación en Materiales Siderúrgicos e INCITEMA, Universidad Pedagógica y Tecnológica de Colombia, Tunja 150003, Boyacaá, Colombia; laura.mejia01@uptc.edu.co (L.G.T.-M.); lais.mujica@uptc.edu.co (L.M.-R.); 3 Departamento de Ingeniería Mecánica, Facultad de Ingeniería, Universidad Tecnológica Metropolitana, Santiago 7800003, Chile; jaguilar@utem.cl; 4 Programa Institucional de Fomento a la Investigación, Desarrollo e Innovación, Universidad Tecnológica Metropolitana, Ignacio Valdivieso 2409, Santiago 8940577, Chile

**Keywords:** stacking fault energy, multicomponent alloys, SQS + DFT, CALPHAD

## Abstract

Multicomponent alloys have attained general interest in recent years due to their remarkable performance. Non-equiatomic alloys with boron addition as an interstitial element are being studied, exhibiting outstanding mechanical properties. In order to estimate the mechanical behavior of potential alloys, thermodynamic and ab initio calculations were utilized in this work to investigate phase stability and stacking fault energy (SFE) for (Fe_50_Mn_30_Co_10_Cr_10_)-xBx (x = 0, 5, 7, 10, and 15 at.%) systems. Thermodynamic experiments revealed two structural variations of borides, M_2_B(C16) with a tetragonal structure and M_2_B(CB) with an orthorhombic structure. Borides precipitate when boron content increases, and the FCC matrix becomes deficient in Mn and Cr. According to ab initio calculations, the presence of boron in the FCC and HCP structures primarily disrupts the surroundings of the Fe and Mn atoms, resulting in an increased distortion of the crystal lattice. This is related to the antiferromagnetic condition of the alloys. Furthermore, for alloys with a low boron concentration, the stacking fault energy was found to be near 20 mJ/m^2^ and greater than 50 mJ/m^2^ when 10 and 15 at.% boron was added. As boron concentrations increase, M_2_B borides are formed, generating changes in the matrix composition prone to fault-induced phase transitions that could modify and potentially impair mechanical properties.

## 1. Introduction

Multicomponent alloys between high-entropy alloys (HEAs) have attained a scientific interest over the last decade due to their remarkable properties, such as a high hardness, ductility, mechanical resistance, thermal stability, wear, and corrosion resistance, to name a few [1,2,3,4,5,6,7,8,9]. These are distinguished by a combination of at least five elements (metallic and non-metallic) with concentrations ranging from 5 to 35 at.%, forming solid solutions (SS) with crystalline structures such as BCC, FCC, and HCP, among others [9,10,11]. In this type of alloys, the effect of high entropy favors the stability of the solid-solution phases, decreasing the tendency for the formation of intermetallic phases, resulting in simpler microstructures with potential applications [12,13,14,15,16,17,18,19,20,21].

The alloy Fe_20_Mn_20_Co_20_Cr_20_Ni_20_, also known as the Cantor alloy, solidifies in a single FCC phase, demonstrating exceptional mechanical properties, strength, and ductility at the same time, showing a notable gap concerning conventional alloys, inclusive at cryogenic temperatures [22,23,24,25]. Currently, the study of HEAs has focused on the addition of non-equiatomic quantities of elements, as well as the incorporation of interstitial elements such as C, N, and B, considering that the hardening of the alloys is influenced by these components, in addition to the main elements that comprise the solid solution [26,27,28,29,30]. Chmielak et al. recently investigated the addition of C and in combination with N as interstitial elements in the CrMnFeCoNi alloy, focusing on its mechanical properties from 77 K to 673 K, microstructure, corrosion resistance, and wear resistance [27]. In comparison to the interstitial free FeMnCoCrNi reference alloy, the addition of C and N results in an increase in yield strength and ultimate tensile strength, and a decrease in the ductility. Additionally, all the systems exhibit a reduction in ductility, strength, and specific fracture energy as the temperature increases. Wear resistance at room temperature was lower than that of austenitic steels, whereas surface corrosion performance was comparable to that of reported austenitic steels [27]. In contrast to the previous results, other studies focused on the addition of small amounts of boron to the equiatomic and non-equiatomic FeMnCoCrNi Cantor alloy [28,29], finding that the material exhibits boron segregation, which has a direct effect on grain size and, as a result, stronger cohesion at the grain boundaries, without resulting in a reduction in ductility. In the multicomponent alloy Fe_50-x_Mn_30_Co_10_CrB_x_ (x = 0, 0.1, 0.66, and 5.4 at.%) [31,32], a dual FCC-HCP phase was obtained from an athermal martensitic transformation (γ→ε), accompanied by the orthorhombic phase type M_2_B(CB) (M = Cr, Fe). When boron was added, microhardness increased from 291 to 445 HV, as well as the wear resistance by nearly 30% above the free boron alloy. This result is attributed to the eutectic strengthening effect and grain refinement of borides, and probably to multiple deformation mechanisms such as martensitic-transformation-induced plasticity (TRIP), twinning-induced plasticity (TWIP), and a dislocation glide, primarily due to an increase in a metastable FCC with the boron addition [31,32].

Behind the mechanical properties of HEAs, austenitic steels, and other FCC engineering materials is the stacking fault energy (SFE). The SFE is a physical property that governs the activation of different deformation mechanisms, mechanical behavior, and the phase transformation of crystalline alloys. SFE represents the energy associated with the tendency to alter the typical stacking fault sequence and build a distinctive type of defects (for example, the dissociation of partial Shockley dislocations), related to the dislocation movement and plastic deformation of metallic materials. In FCC metals, SFE is determined with the dissociation distance of partial dislocation in the {111} <110> slip system, and the modification in the stacking sequence results in a local stacking fault structure such as hexagonal-close-packed (HCP) nuclei. When SFE values are below 40 mJ/m^2^, it is commonly considered that martensitic-transformation-induced plasticity (TRIP) and deformation twinning are dominant, whereas a dislocation glide is typically present in materials with a high SFE [33]. As a result, SFE estimation is a powerful parameter for predicting and studying the mechanical behavior of multicomponent alloys.

The theoretical prediction as well as the experimental estimation of the SFE is not straightforward. Transmission electron microscopy (TEM) and X-ray diffraction (XRD) are utilized to determine the SFE by measuring the spacing between dissociated partial dislocations or obtaining the stacking fault probability from the mean square micro-strain ε^2^, respectively [34,35,36,37]. Thermodynamic calculations have been used to estimate SFE for various alloys using the Olson and Cohen equilibrium thermodynamic formalism [38,39,40]. Ab initio calculations using solid solution modelling at T = 0 K have also been used to determine SFE, allowing the atomistic comprehension of the mechanical behavior [41,42,43,44,45,46,47,48,49]. For example, combining experimental and theoretical calculations, the mechanical properties and SFE of the Cantor alloy and four non-equiatomic derivatives were examined, yielding values in the range of 30 ± 5 mJ/m^2^ [50]. Fe_40-x_Mn_20_Co_20_Cr_20_Ni_x_ (x = 0–20 at.%) HEAs were recently studied using an ab initio design in relation to the HCP-FCC energy differences (ΔE_HCP-FCC_). The results revealed a substantial relationship between the HCP-FCC phase stability and Ni content, implying that the Fe_34_Mn_20_Co_20_Cr_20_Ni_6_ HEA had the highest strength [51,52]. Based on the foregoing, it is possible to study and predict the mechanical behavior using the SFE, integrating structural and thermodynamic characteristics, which provide a more complete understanding of the materials and, as a result, facilitate the design of promising alloys in studies related to the optimization of properties such as ductility, malleability, and hardness [42,43,49,51,52,53,54,55,56,57,58,59].

In this work, a thermodynamic and ab initio alloy design is proposed, based on the system Fe-Mn-Co-Cr with the addition of B as an interstitial element in 0, 5, 7, 10, and 15 at.%, with the aim of comparing and predicting the microstructural results of the Fe-Mn-Co-Cr alloy with a low boron content (0 and 5 at.%), and providing an insight on the structural features and mechanical behavior when 7, 10, and 15 at.% of boron is added. Empiric phase rules, phase diagrams, thermodynamic stability, and stacking fault energy make up part of this study. The final objective was to understand how the boron addition affects the relationship of structure–mechanical properties. In this context, the design of HEAs with boron addition is a promising concept that provides a solution to the abrasive wear of equipment and deterioration problems in surface engineering uses [60,61].

## 2. Materials and Methods

### 2.1. Phase Prediction with Empiric Parameters

The concentration of the components, along with specific physicochemical factors, are critical in the investigation of HEA formation. Parameters such as the entropy and enthalpy of mixing, as well as lattice distortion, are studied for a better understanding and prediction of their behavior [62,63].

According to Ludwig Boltzmann, who defined the configurational entropy, the more components there are, the more alternative configurations exist, and hence the entropy increases. Thus, for a random n-component solid solution, the ideal configurational entropy per mole is given by the following [54]:

(1) $$∆S=-R\sum_{i=1}^{n}C_{i}lnC_{i}$$

where *R* represents the gas constant (8.314 J/mol K), $C_{i}$ corresponds to the atomic percentage of component i, and n is the number of alloy components.

The enthalpy of mixing is the amount of energy that a system exchanges with its surroundings, and is expressed as

(2) $$∆H=\sum_{i=1,\;j\neq 1}^{n}\Omega_{ij}C_{i}C_{j}$$

where $\Omega_{ij}$ is the mixing enthalpy of binary alloys, with $C_{i}$ and $C_{j}$ as the mole fractions of components i and j, respectively [54]. The enthalpy of mixing defines the distribution of atoms in the solid solution (SS), which is grouped if $∆H_{mix}>0$ or dispersed if $∆H_{mix}<0$ [9].

The previous parameters can be employed in the variable *Ω* that relates the effects of the enthalpy and entropy of mixing to predict the formation of a solid solution phase and is expressed as

(3) $$\Omega =\frac{T_{m}∆S_{mix}}{\left|∆H_{mix}\right|}$$

(4) $$\;T_{m}=\sum_{i=1}^{n}C_{i}{\left(T_{m}\right)}_{i}$$

where $T_{m}$ corresponds to the average melting temperature of the set of alloy components; likewise, ${\left(T_{m}\right)}_{i}$ denotes the inherent melting point of each component [64,65].

Similar electro-negativities of the solute and solvent are associated with the formation of solid solutions. Nevertheless, a significant difference indicates a tendency for the formation of intermetallic phases. This difference is known as

(5) $$∆\chi =\sqrt{\sum_{i=1}^{n}C_{i}{\left(X_{i}-X_{av}\right)}^{2}}$$

where n is the number of elements, $C_{i}$ and $X_{i}$ are the composition, and the Pauling electronegativity of the alloy is expressed as $X_{av}$ [64].

The solute–solvent atomic size difference is a crucial factor in predicting solid solution formation in HEA. A difference of less than 15% indicates its formation. This difference is denoted as follows:

(6) $$\delta =\sqrt{\sum_{i=1}^{n}C_{i}}{\left(1-\frac{r_{i}}{\bar{r}}\right)}^{2}$$

(7) $$\bar{r}=\sum_{i=1}^{n}C_{i}r_{i}$$

where $\bar{r}$ is the average of the atomic radii of the elements of the system, $r_{i}$ is the atomic radius of the element i, and $C_{i}$ is the atomic percentage of the element i in the HEA.

HEAPS software V1.0 (High Entropy Alloy Prediction Software) was used to determine these parameters [66]. The parameters were classified into various criteria, which are listed in Table 1.

### 2.2. Thermodynamic CALPHAD Calculations: Phase Diagram Prediction

The CALculation of PHAse Diagrams (CALPHAD) approach was used in conjunction with Thermo-Calc software version 2023 and the TCEF9 database. For the (Fe_50_Mn_30_Cr_10_Co_10_)-xBx system, equilibrium calculations were carried out to estimate the phase diagram as well as the molar fraction of stable phases as a function of temperature and molar fraction of elements in phases as a function of temperature.

The Olson–Cohen model for the intrinsic SFE in FCC metals and alloys was used to estimate the stacking fault energy from a thermodynamic approach [39], complemented by the model proposed by Hirth, where a stacking fault in an FCC crystal structure consists of a thin-layer HCP phase [67]. The most significant contribution is the difference in Gibbs free energy ($∆G_{C}^{\gamma \to \varepsilon })\;$ between the *ε*-HCP and *γ*-FCC phases. As a result, the *SFE* (*γ_isf_*, mJ/m^2^) can be expressed as follows:

(8) $$SFE\;\left(\gamma_{isf}\right)=2.\frac{4}{\sqrt{3.a^{2}.N_{A}}}.\;∆G_{C}^{\gamma \to \varepsilon }+2.\sigma^{\frac{\gamma }{\varepsilon }}$$

where $∆G_{C}^{\gamma \to \varepsilon }\;$ is the molar Gibbs energy difference in the phase transformation between the austenite  γ and the ε-martensite. At 298.15 K and 1 bar, metastable phase calculations in terms of Gibbs free energy as a function of the composition were performed using Thermo-Calc software and the TCFE9 database, considering the matrix composition from the equilibrium calculations (affected by boride formation), suspending all phases except for FCC and HCP. The interfacial energy, which ranges from 0 to 10 mJ/m^2^, is denoted with the term $\;\sigma^{\gamma /\varepsilon }$. Based on data provided for a comparable, an average value of 8 mJ/m^2^ was assumed [37,68,69]. The expression $\frac{4}{\sqrt{3.a^{2}.N_{A}}}$ describes the molar surface density along {1 1 1 }  planes, where a is the lattice parameter of the alloys specified in references [32]. $\;N_{A}$ is Avogadro’s number, and the integer value 2 denotes the number of densely packed planes in the HCP ε-martensite phase.

### 2.3. Ab Initio DFT Solid Solution Modelling

Solid solution modelling was applied to obtain a random structure with the optimal chemical disorder arrangement [43]. This was performed using the special quasi-random structures (SQS) methodology [70,71,72,73,74,75] implemented in an alloy theoretic automated toolkit (ATAT) program [76]. An objective function was minimized, taking into account (i) a supercell size of 60 atoms, (ii) a chemical composition of (Fe_50_Mn_30_Cr_10_Co_10_)-xBx (x = 0, 5, 7, 10, and 15 at.% associated with alloy-B0, alloy-B5, alloy-B7, alloy-B10, and alloy-B15, respectively), and (iii) a third nearest-neighbor distance for FCC and HCP structures ranging from 3.0 to 6.0 Å. Afterwards, 1 and 2 boron atoms were added as interstitial atoms in octahedral sites into Fe_30_Mn_18_Co_6_Co_6_ and Fe_27_Mn_15_Co_9_Cr_9_ alloys, according to the heat of mixing reported for the metal-boron binary [77]. It should be noted that the compositions of the studied alloys, (Fe_50_Mn_30_Cr_10_Co_10_)-xBx (x = 0, 5, 7, 10, and 15 at.%), will be addressed considering the metal alloy as 100, 95, 93, 90, and 85% of the solid solution, respectively. When the boron level exceeded 10 at.%, the Fe, Mn, Co, and Cr amounts fluctuated dramatically. Solid solution structures for the intermetallic compounds (Cr, Fe)_2_B (M_2_B_CB_) and (Fe,Cr)_2_B (M_2_B_C16_) with an orthorhombic and tetragonal crystalline structure, respectively, were also obtained to complement the discussion. All the exact compositions are listed in Table 2:

A Vienna Ab-initio Simulation Package (VASP, version 6.2) [78,79] was used to perform DFT periodic calculations with the Perdew–Burke–Ernzerhof (PBE) exchange–correlation functional, which has been widely reported for extended systems [80,81,82]. The valence electrons are extended in a plane waves basis set for each metal, and the core electrons are characterized by the projector augmented wave (PAW) pseudopotential. For the (5 5 5) K-point mesh, the Monkhorst–Pack sampling of the Brillouin zone was used. This mesh was chosen based on the optimal trade-off between the accuracy and the computational cost that had previously been evaluated [83]. ISIF = 3 was used to maximize all FCC and HCP supercells, cell shape, and cell volume. The plane waves’ energy cut-off was set to 500 eV, the self-consistent field (SCF) tolerance was 1 × 10^−6^ eV, and the geometry relaxation was considered as convergent when the energy difference from the previous optimization step was less than 1 × 10^−5^ eV. To account for the magnetic properties of the studied alloys, the co-linear spin correction energy (ISPIN = 2) was included in the optimized geometry. For each atom, we specified the initial magnetic moment: Fe (S = 1), Mn (S = −1.5), Co (S = 1.5), and Cr (S = 3) for a “ferromagnetic” state (FM) and Fe (S = −1), Mn (S = −1.5), Co (S = −1.5), and Cr (S = −3) for an antiferromagnetic state (AFM). Furthermore, a spin state (AFM-FM = PM) was evaluated, considering half of the moments as up and the other moments as down.

To investigate the phase stability and solubility of boron in the alloys, the formation enthalpy $({\Delta H}_{f})\;$ [9,10] was calculated using the total energy of the optimal alloy supercell and the weighted energies of each element in their most stable magnetic state as follows:

(9) $$∆H_{f\left(A_{m},B_{n},C_{o},D_{p},E_{q}\right)}=E_{\left(A_{m},B_{n},C_{o},D_{p},E_{q}\right)}-\frac{1}{m+n+o+p+q}\left({\mathrm{mE}}_{\left(A\right)}+{\mathrm{nE}}_{\left(B\right)}+{\mathrm{oE}}_{\left(C\right)}+{\mathrm{pE}}_{\left(D\right)}+{\mathrm{qE}}_{\left(E\right)}\right)$$

The SFE was obtained using Stocks et al.’s [48,84] axial interaction model. Based on this approach, the SFE for the martensitic transformation can be estimated by taking into account the interactions of the (111) layer up to the nearest neighbor (ANNI model), which is defined in terms of the total energy of FCC and HCP structures, as well as the area of the (111) plane (*A*). Thus, the SFE can be expressed as

(10) $$SFE_{ANNI}\;=\frac{E_{hcp}-E_{fcc}}{A}$$

The SFE for the phase transformation between the intermetallic compounds (Cr, Fe)_2_B and (Fe,Cr)_2_B was computed using this definition, considering the total energy of orthorhombic and tetragonal structures, as well as the area of the (001) plane (A) in relation to that reported by Goldfarb and co-workers [85]. Finally, it is important to note that this equation predicts the SFE exclusively through ab initio calculations, enabling the rationalization of strain-hardening behavior based on the structural and physicochemical properties.

## 3. Results

### 3.1. Empiric Formation Phase Rule Analysis

The enthalpy of mixing, entropy of mixing, valence electron concentration, electronegativity, distortion parameter, ratio of entropy/enthalpy contribution, and melting temperature of the alloys (Fe_50_Mn_30_Cr_10_Co_10_)-xBx (x = 0, 5, 7, 10, and 15 at.%) were determined (see Section 2.2). Table 3 shows that the distortion parameter (δ_r_) ranges between 3.83 and 13.96%, which can be connected with the formation of stable solid solutions, because it has been reported that multiphase/intermetallic HEA takes place when 1 ≤ δ_r_ ≤ 13.5%. Furthermore, the electronegativities are similar, which support the solid solution formation. Additionally, the enthalpy of mixing revealed a random distribution of atoms since $∆H_{mix}<0$. These parameters increase as the boron content increases, indicating that this element forms part of the solid solution as an interstitial atom. VEC parameter values are in the same range as those published for Fe_50_Mn_30_Co_10_Cr_10_B_x_ (x = 0, 0.1, 0.66, and 5.4 at.%) [32]. These values are solely applied to the boron-free alloy, which exhibited both FCC and BCC phases, because it has been shown that both FCC and BCC phases are stable at 6.78 ≤ VEC ≤ 8.0.

Table 4 describes the criteria for the formation phase of alloy-B0 through alloy-B5 in HEAPS software. Based on the MC1 formation criteria, it is possible to observe that raising the boron level from 5 to 10 at.% can result in the formation of either an intermetallic compound (IM) or a bulk metallic glass (MBG). Similar results were found using the MC2 criterion, which compared the parameter Ω (which relates both ∆S and Tm in relation to ∆H) with the variable δ_r_. Therefore, the findings are consistent with previous research that discovered the formation of compounds such as iron and chromium borides (M_2_B) [32,86].

The MC7 criterion suggested that an intermetallic phase would form without and with the boron addition. However, it is not impossible because the mixing enthalpy is negative, indicating the formation of stable solution solids, thus being a mistake in the HEAPS software criterion. Moreover, for alloy-B0 (Fe_50_Mn_30_Co_10_Cr_10_), the phases BCC, FCC, and HCP in solid solutions were determined experimentally, confirming the error mistake in this criterion [32]. The MC7 criterion solely examines the relationship of T over Tm-δ_r_-∆H_mix_, and does not include the ∆S of the mixture. This variable is particularly relevant since boron interacts differently in the system depending on the elements with which it is coordinated, affecting the microstates that comprise the overall system state. Finally, the IMF1 criterion evaluated using ∆X^P^ suggests the formation of topologically compact phases (TCP), which is attributed to an unusually robust structural state, which would be interesting to explore in future investigations.

### 3.2. Phase Diagram Prediction for (Fe_50_Mn_30_Cr_10_Co_10_)-xBx (x = 0, 5, 7, 10, and 15 at.%)

**Figure 1:**
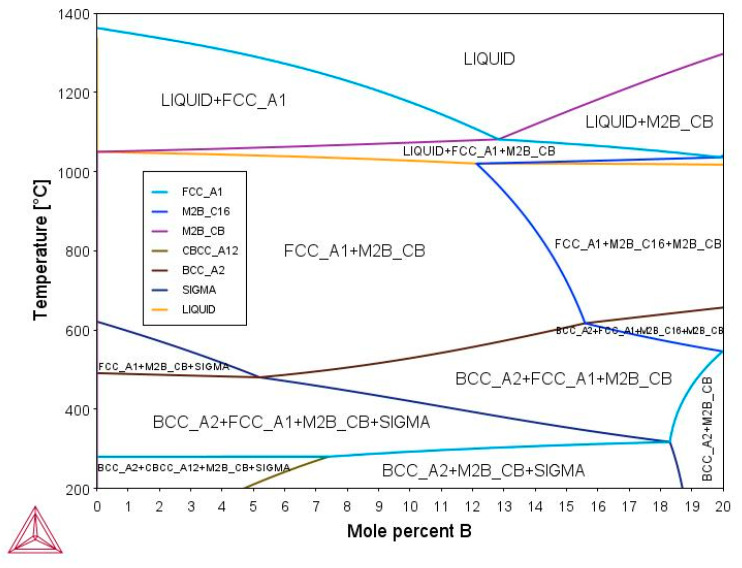
Pseudobinary phase diagram for (Fe_50_Mn_30_Cr_10_Co_10_)-xBx system.

The pseudobinary phase diagram was calculated as a function of the boron content (at.%) (Figure 1), under thermodynamic equilibrium conditions. According to the isoplethal section, a stable fcc austenitic structure and M_2_B borides (M denotes transitional metals) are formed from the liquid phase to 1200 °C until solidification. Besides the austenitic matrix, two structural variants of M_2_B-type borides can be present in the microstructure, M_2_B(C16) having a body-centered tetragonal structure (Strukturbericht notation, C16; space group, I4/mcm) and M_2_B(CB) with a face-centered orthorhombic structure (Strukturbericht notation, Cb; space group, Fddd). M_2_B(CB) borides are found as a primary phase at boron contents lower than 15 at.%; meanwhile, M_2_B(C16) boride precipitates when the boron content is larger than 15 at.%, as a solid-state transformation under thermodynamic equilibrium conditions. Thus, one of the most important features of the phase diagram is the change in crystalline structure of the M_2_B borides from orthorhombic to tetragonal as the content of the interstitial element increases from 15 to 20 at.%. At temperatures below 600 °C, the austenitic matrix could decompose to a bcc ferritic matrix and intermetallic sigma phase.

**Figure 2:**
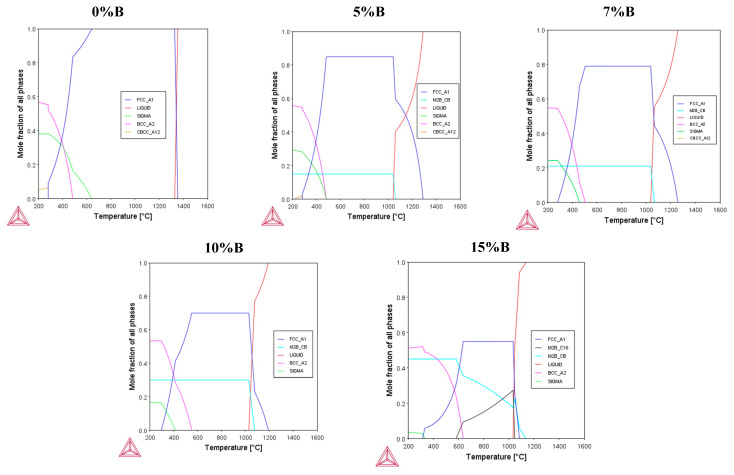
Molar fraction of stable phases as a function of temperature.

Figure 2 illustrates a one-axis calculation of the molar fraction of stable phases for (Fe_50_Mn_30_Cr_10_Co_10_)-xBx (x = 0, 5, 7, 10, 15, and 20 at.%) as a function of temperature. At a boron content of 0 at.%, an FCC austenitic phase begins to form at a temperature of around 1300 °C. Additionally, at low temperatures, the sigma and BCC phases can occur. In the case of alloy-B0 fabricated with laser cladding, FCC and BCC were formed under conditions out of the thermodynamic equilibrium [32]. Nevertheless, the temperature–time process conditions, especially the cooling rate, are what determine whether the BCC phase is present or not. If the boron content increases, there is a significant decrease in the FCC phase. For boron contents of 5, 7, and 10 at.%, an increase in M_2_B(CB) is generated with FCC/boride ratios of 0.9/0.1, 0.8/0.2, and 0.7/0.3, respectively. Furthermore, the existence of M_2_B(C16) borides was undeniably present for boron contents of 15 and 20 at.%. Thus, it can be seen that the ratio of M_2_B(CB)/M_2_B(C16) is 0.6/0.4 when the boron content is 15 at.% and 0.3/0.7 when the boron content is 20 at.%. Therefore, the amount of tetragonal boride increases when the boron content is increased from 15 to 20 at.%, while the orthorhombic boride decreases and the FCC phase gradually disappears, which is related to the phase transformation into the two types of M_2_B borides.

**Figure 3:**
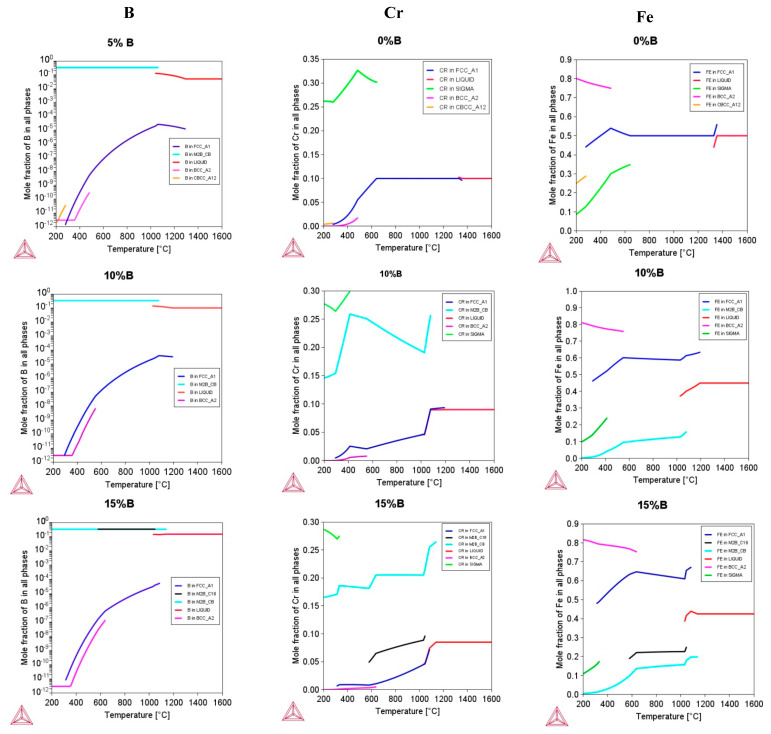
Molar fraction of elements in phases as a function of temperature.

Figure 3 shows the Cr, Fe, and B distribution in the phases with varying boron contents. For the boron-free system, it can be seen that Cr is mainly distributed in the FCC phase, while with the addition of boron, the FCC matrix is depleted, and a greater proportion of Cr is found in borides, mainly in M_2_B(CB). This indicates that the formation of Cr-rich borides is possible, as was also observed in similar alloys produced with arc-melting and laser cladding [31,32]. Fe is mainly distributed in the FCC phase for the free boron system. Thus, small amounts of iron are present in the M_2_B(CB) boride when the boron content increases. It is noted that Fe can be found in borides with a larger B addition (15 at.%), in this case, mostly in the M_2_B(C16) type. Interestingly, these changes were observed in boron-doped alloys [86].

Finally, at a boron percentage of 5 at.%, it is noticed the M_2_B(CB) boride predominantly contains the greatest amount of boron, whereas the FCC matrix decreases significatively in the boron content. Thus, as the boron percentage increases to between 10 and 15 at.%, the presence of borides becomes more stable, and the matrix retains a low boron content.

Regarding non-equilibrium calculations on the stability of the FCC structure in relation to the deformation mechanisms and mechanical properties, SFE values were obtained with the CALPHAD method, as are shown in Table 5. An increase in boron content modifies the Gibbs free energy of the FCC and HCP lattice; in all cases, the energy associated with the FCC phase has lower values, indicating that it is more stable than the HCP phase. SFE increases with boron addition. For alloy-B0 and alloy-B5, the values are in the range of others reported [50]. Nevertheless, a significant variation of approximately 20 mJ/m^2^ is observed for the alloy containing 7 at.% boron, whereas the variation of the SFE is approximately 4 mJ/m^2^ for higher boron contents. This suggests a substantial change in the deformation mechanisms and in the mechanical properties of the alloy with a 7 at.% boron content.

### 3.3. Structure, Stability, Magnetic Role, and SFE for the Design Alloys (Fe_50_Mn_30_Cr_10_Co_10_)-xBx (x = 0, 5, 7, 10, and 15 at.%)

**Figure 4:**
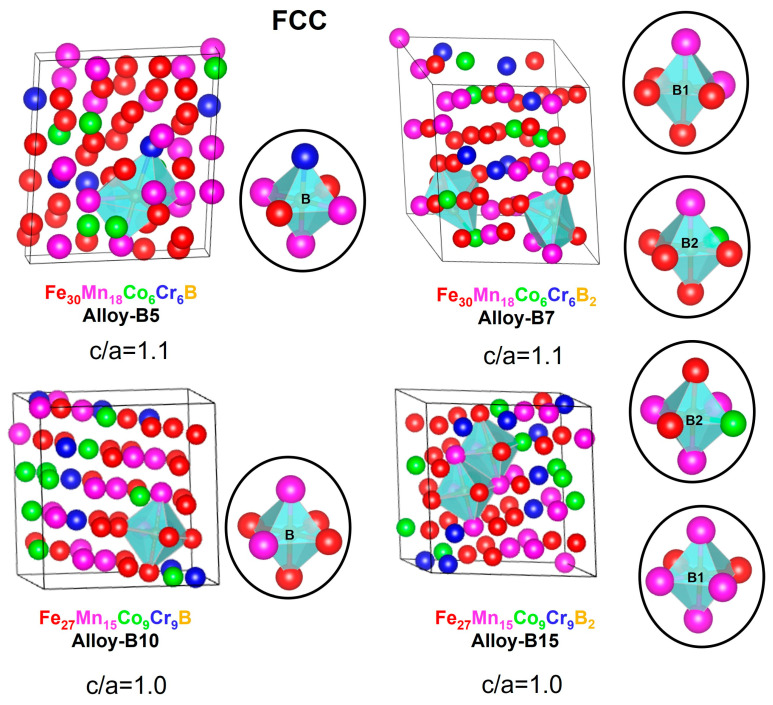
Structural parameters and octahedral cavity for the FCC alloys (Fe_50_Mn_30_Cr_10_Co_10_)-xBx (x = 0, 5, 7, 10, and 15 at.%) obtained with SQS + DFT. All values given are in Angstrom (Å). Red spheres = iron, magenta spheres = manganese, green spheres = cobalt, blue spheres = Chromium and yellow spheres = boron.

**Figure 5:**
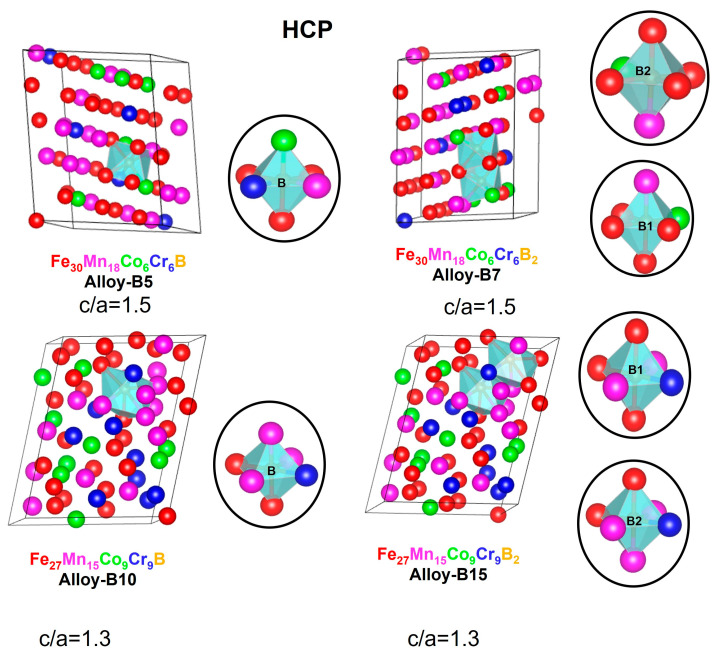
Structural parameters and octahedral cavity for the HCP alloys (Fe_50_Mn_30_Cr_10_Co_10_)-xBx (x = 0, 5, 7, 10, and 15 at.%) obtained with SQS + DFT. All values given are in Angstrom (Å). Red spheres = iron, magenta spheres = manganese, green spheres = cobalt, blue spheres = Chromium and yellow spheres = boron.

Figure 4 and Figure 5 depict the 60-atom optimized supercells for the FCC and HCP crystalline structures of the alloys (Fe_50_Mn_30_Cr_10_Co_10_)-xBx (x = 0, 5, 7, 10, and 15 at.%). The lattice parameters are closest to a perfect FCC and HCP structure with slight distortions characteristic of HEAs. In FCC, alloy-B5 and alloy-B10 are more ordered systems than alloy-B7 and alloy-B15, indicating that the larger boron addition in the supercell may affect the entropy of the systems. In FCC and HCP, the lattice parameters of alloy-B10 and alloy-B15 decrease slightly, which is associated with a lower Fe and Mn content compared to alloy-B5 and alloy-B7.

According to the mixing enthalpies reported for binary metal-boron systems (∆ Hmix M-B = −32 (MnB), −31 (CrB), −26 (FeB), and −24 (CoB) kJ/mol), manganese has the strongest affinity for boron, followed by chromium, iron, and cobalt [77]. For the FCC and HCP structures, it can be clearly observed that the boron prefers to form octahedral interactions with manganese and iron elements. Therefore, these atoms play a stabilizing role in the formation of alloys of this type.

Moreover, the prediction of the martensitic transformation γ→ε was considered. In this regard, the principal diagonal of the FCC structures was compared to the c-parameter of HCP. The FCC principal diagonal in Fe_30_Mn_18_Co_6_Cr_6_B is 11.78 Å, while the c-parameter of HCP is 10.77 Å. Similar outcomes were observed for all the alloys modeled with the principal diagonal between 11.78 and 12.66 Å and the c-parameter between 10.48 and 10.77 Å. Consequently, the models applied in this work are structurally appropriate for analyzing martensitic transformations.

In order to compare the atomic distortions in the crystal lattice caused by the addition of boron, the root mean square deviation (RMSD) was calculated. According to Table 6, the average distortion of all elements in FCC phase lattice structures is greater than in HCP (alloy-B5 versus alloy-B7 and alloy-B10 versus alloy-B15). This result is attributed to the fact that the atoms of the FCC phase structures tend to deform into HCP phase structures. In terms of distortions per atom, the Cr and Co atoms are the most distorted in the FCC structures, while the Mn and Fe atoms are the most distorted in the HCP structures. Therefore, the results of the lattice distortions indicate that when boron is added, a greater distortion is generated, stabilizing interactions that could contribute to reducing the Gibbs free energy and achieving a solid solution with a low Gibbs free energy.

The values listed in Table 7 were examined in order to comprehend the stability of the alloys. The HCP phase was found to be more stable than the FCC phase in alloy-B0, alloy-B5, and alloy-B7, which is consistent with experimental studies of the Fe_50-x_Mn_30_Cr_10_Co_10_B_x_ (x = 0 and 5 at.%). This study revealed that the predominant relative HCP phase composition was 85 and 64 at.% for the addition of 0 and 5 at.% of boron, respectively [32]. Moreover, the phase energy difference (ΔE_HCP-FCC_) indicates that 5.75 eV and 7.11 eV are required when 5 and 7 at.% boron is added, respectively. On the other hand, the energy values for alloy-B10 and -B15 had an opposite trend, indicating that the FCC phase is more stable than the HCP phase.

This conclusion is consistent with the SFE results obtained using the CALPHAD approach, which shows that the SFE rises due to the increased stability of the FCC phase against the HCP transformation. Thus, boride formation causes a significant alteration in the behavior of the FCC solid solution of alloys with a high boron content.

The enthalpy of formation for each alloy was estimated (Table 8). The values are most closely related to −14.0 kJ/mol. These results show that non-equiatomic alloys with boron additions are 6.0 kJ/mol more stable than the Fe_20_Mn_20_Cr_20_Co_20_Ni_20_ Cantor alloy (−8.43 kJ/mol) [50]. A similar tendency for total energy was found. The enthalpy of formation for HCP structures is lower for alloy-B0 and -B5, while it is higher for alloy-B7 to alloy-B15. Again, this suggests that when the boron content is high, the alloys exhibit a different physicochemical behavior. To scrutinize this topic, the enthalpy of formation for the intermetallic compounds, (Cr, Fe)_2_B and (Fe,Cr)_2_B, was calculated to be −23.0 and −23.7 kJ/mol, respectively. These values are considerably greater than the enthalpy of formation of the solid solution, which favors the formation of intermetallic compounds.

The magnetic corrections to the energy were calculated using spin-polarized calculations with an initial specified magnetic moment for each atom (Table 7). At T = 0 K, the alloys exhibited a “ferromagnetic” state. In this state, Cr, Fe, and Co have parallel spin states whereas Mn is exclusively antiparallel. As has been widely explicated, the Mn content plays a critical role in t magnetic stabilization [87,88]. Moreover, it is important to highlight that the boron addition can also be responsible for the magnetic behavior because a paramagnetic state has been reported for similar alloys to the CrMnFeCoNi Cantor alloy at room temperature [89]. Therefore, the boron addition favors a magnetic transformation at T = 0 K.

Table 9 shows the SFEs generated from the axial interaction model represented in Equation (10). The values for alloy-B0 to alloy-B7 range from 18.34 to 23.0 mJ/m^2^—consistent with the Cantor alloy (17–25 mJ/m^2^) [50]. The results showed that the SFEs increased as the B content increased by 7 at.%, implying that the presence of B generated a hardness that promoted the dissociation or segregation via twinning-induced plasticity (TWIP) and dislocation slip deformation mechanisms, affecting the SFE. These deformation mechanisms have an origin in the Fe-B and Mn-B short-range order interactions represented in an FCC solid solution’s octahedral cavity. Nevertheless, when the boron addition is greater than 10 at.%, the SFE decreases significantly. Therefore, it is suggested that the martensitic transformation is hindered due to the impoverishment of Cr and Fe atoms from the matrix in terms of participating in the formation of the M_2_B(CB) borides, which could be responsible for the lower SFE.

**Figure 6:**
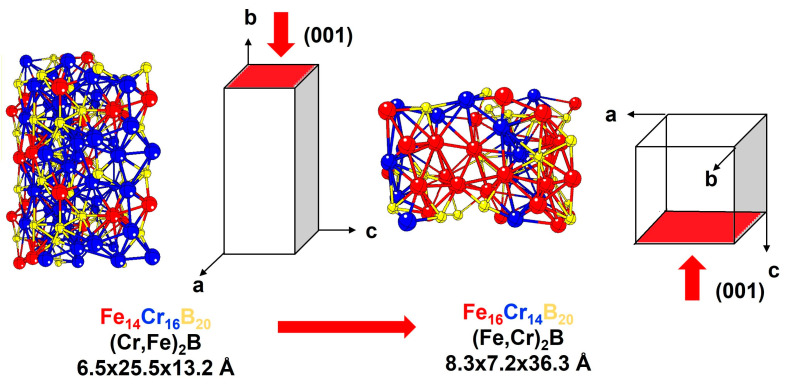
Structural parameters of a 3 × 3 × 3 cell of orthorhombic (Cr,Fe)_2_B and tetrahedral (Fe,Cr)_2_B.

These SFE results are corroborated with the CALPHAD calculations, which indicate that the fcc matrix coexists with M_2_B(CB) and M_2_B(C16) borides in alloy-B10 and alloy-B15, respectively. These boride types have been experimentally identified with XRD [32] and HR-TEM [86], suggesting that M_2_B(CB) is a (Cr,Fe)_2_B that begins to transform into (Fe,Cr)_2_B if the Cr content exceeds the solubility limit. This transformation is a “fault-induced phase transformation” [85,90,91]. In this direction, we build and optimize, with SQS + DFT, a model for the orthorhombic (Cr,Fe)_2_B and tetragonal (Fe,Cr)_2_B (Figure 6). The phase energy difference (ΔE_ORT-TETRA_) was determined to be 14.35 eV. Additionally, the SFE measured at 46.37 mJ/m^2^. For alloy-B10 and alloy-B15, it is suggested that the SFE should be the sum of the SFE resulting from the martensitic transformation (SFE_ANNI_) and the SFE resulting from the boride phase transformation (SFE_M2B_).

## 4. Discussion

The thermodynamic parameters, CALPHAD method, and SQS + DTF calculations allowed for estimating the phase prediction and SFE results of the multicomponent (Fe_50_Mn_30_Co_10_Cr_10_)-xBx (x = 0, 5, 7, 10, 15, and 20 at.%) system alloy. Boron has an evident influence on the physical and chemical parameters of the HEAs proposed. Multiple phases are expected based on thermodynamic prediction when boron is added to this alloy. It is essential to highlight that a single phase is stable at high temperatures in the boron-free alloy. At higher concentrations of this interstitial element, borides form and the fcc matrix is depleted in Cr and Mn, which contributes to the stabilization of borides as an equilibrium phase transformation.

On the other hand, the hcp phase is not enclosed in the equilibrium calculations (Figure 1, Figure 2 and Figure 3), due to the fact that it is related to a non-equilibrium phase transformation caused by the high cooling rate during the solidification process in metastable conditions. The free Gibbs energy difference of the FCC (γ) and HCP (ε) phases with respect to the martensitic transition γ-ε is directly connected to the tendency to produce the HCP phase (Table 5). The larger the $\Delta G^{\gamma \to \varepsilon }$, the less likely an ε- or α-martensitic structure will be formed during the deformation of the thermal shock.

Considering the empiric formation phase rule analysis utilizing HEAPS, the best-fit phase prediction criteria are MC1, MC2, MC7, and IMF1. Experimentally, a dual phase (FCC + HCP) was obtained for this boron-free alloy, where HCP is caused by both a thermal martensitic-like partial-transformation-induced FCC phase and a high cooling rate [28,31,32]. For the IMF1 and VEC conditions, these characteristics would fit the sigma phase illustrated in Figure 3, which is often Cr-and-Mn-rich [83] but has not been observed experimentally because it is driven by diffusion processes that require extended heat treatments.

It is well known that the phase stability is determined with the competition between ΔH and TΔS, minimizing the Gibbs free energy. Nevertheless, as demonstrated for this system, a multicomponent alloy with distinct phases can be formed based on the boron addition. Thus, the stability of the system is determined not only by entropy but also by the enthalpy of phase formation, particularly for intermetallic compounds.

We observe that the SFE rises as the amount of boron increases. In accordance with the SFE value reported for the Cantor alloy, the SFE values for alloys with a minimal boron content (alloy-B0 to alloy-B7) fall between 20 and 23 mJ/m^2^. With an increasing boron content (alloy-B10 to alloy-B15), the SFE increases significantly to 50–60 mJ/m^2^.

In the first instance, the stacking fault sequence and the mobility of defects and dislocations are affected by the presence of boron in the solid solution and the formation of boride, which acts as an impediment to the gliding of Shockley partial dislocations.

The increased SFE can be explained with the formation of two boride types, orthorhombic and tetragonal, which undergo a “fault-induced phase transformation” as the boron content rises. Thus, it is suggested that the martensitic transformation is blocked as a result of Cr and Fe atoms in the matrix that becomes depleted of the solid solution in order to participate in forming the borides, thereby influencing the matrix composition. Consequently, this additional transformation impedes the formation of new stacking faults and the movement of dislocations.

Considering the applications of the alloys proposed in this study, particularly in terms of hardness, wear, and corrosion resistance, the following aspects can be considered. It is expected that the hardness of the matrix (either FCC or FCC-HCP) will be greater in systems with a low SFE (alloy-B0 and -B5). Despite this behavior, the overall hardness of the system can increase due to higher concentrations of borides, which are hard phases (alloy-B10 and -B15). In this regard, the combination of hard phases accompanied by a tough and ductile matrix can produce promising results in terms of the wear resistance of the alloys designed. On the other hand, the impoverishment of chromium from the FCC matrix towards the borides could reduce the corrosion resistance of the alloys due to a lack of passivation capability. In this respect, it is important to find a balance between the matrix mechanical properties (SFE), the amount of borides (hard phases), and the chromium content of the matrix for an optimal performance of equipment subjected to wear and corrosion. Future research should be carried out in the study of the phase stability from experimental approaches involving heat treatments.

## 5. Conclusions

For (Fe_50_Mn_30_Cr_10_Co_10_)-xBx (x = 0, 5, 7, 10, and 15 at.%), thermodynamic and ab initio calculations were conducted to determine the effect of boron composition. When boron is added, empiric phase rules and a thermodynamic analysis predict the formation of intermetallic compounds. Specifically, the phase diagram at a solidification temperature reveals the presence of an FCC phase and two types of borides, one orthorhombic at boron contents greater than 5 at.% and one tetragonal at boron contents greater than 15 at.%. In this last case, the ratio of orthorhombic/tetragonal is 0.6/0.4. In addition, theoretical calculations were performed to examine the phase stability and stacking fault energy in the alloys. The results showed that when boron is introduced, the lattice distortion increases due to the Fe-B and Mn-B short-range order interactions into the octahedral cavities of the structures, which can give rise to a low formation enthalpy for the solid solutions. Nevertheless, the formation enthalpy of the intermetallic compounds was more stable at 9 kcal/mol than the solid solutions, demonstrating that the production of intermetallic compounds is more favorable when boron is present. Regarding the stacking fault energy of the alloys, it was determined to be closest to 20 mJ/m^2^ for alloys with a minimal boron content and greater than 50 mJ/m^2^ when 10 and 15 at.% of boron is added. According to these findings, borides undergo a “fault-induced phase transformation”.

## Figures and Tables

**Table 1:** Criteria and parameters used to determine the stability of the solid solution.

Criteria	Parameters	Range
MC1	δr	0.5%<δr<6.5%
	$∆H_{mix}$	−17.5 kJ/mol<∆Hmix<−5 kJ/mol
MC2	Ω	Ω≥ 1.1
	δr	δr≤ 6.6%
MC7	$T/T_{m}$	$0.9<T/T_{m}$
	$∆H_{mix}$	−15 kJ/mol<∆Hmix<5 kJ/mol
	δr	δr ≤ 6.6%
	$T/T_{m}$	$0.5\;\leq \;T/T_{m}<0.9$
	$∆H_{mix}$	$∆H_{mix}\geq -7.5\;\mathrm{kJ}/\mathrm{mol}$
	δr	δr ≤ 3.3%

**Table 2:** Exact compositions and supercell compositions (at.%) corresponding to (Fe_50_Mn_30_Cr_10_Co_10_)-xBx (x = 0, 5, 7, 10, and 15 at.%).

Nomenclature	Exact Composition (at.%)	60 Atoms’ FCC and HCP Supercell Composition
Alloy-B0	Fe_50_Mn_30_Co_10_Cr_10_	Fe_30_Mn_18_Co_6_Cr_6_
Alloy-B5	Fe_48_Mn_27_Co_10_Cr_10_B_5_	Fe_30_Mn_18_Co_6_Cr_6_B
Alloy-B7	Fe_48_Mn_27_Co_9_Cr_9_B_7_	Fe_30_Mn_18_Co_6_Cr_6_B_2_
Alloy-B10	Fe_45_Mn_27_Co_9_Cr_9_B_10_	Fe_27_Mn_15_Co_9_Cr_9_B
Alloy-B15	Fe_42_Mn_25_Co_9_Cr_9_B_15_	Fe_27_Mn_15_Co_9_Cr_9_B_2_

**Table 3:** Physical and chemical parameters for the study of the phase rules of the HEAs.

Nomenclature	∆S_mix_ (J/mol*K)	∆H_mix_ (kJ/mol)	VEC	∆X^P^	δr (%)	Ω	Tm (K)
Alloy-B0	9.71	−0.92	7.60	0.132	3.83	18.54	1756
Alloy-B5	10.94	−6.19	7.38	0.143	8.75	3.17	1792
Alloy-B7	11.02	−8.07	7.29	0.148	10.06	2.46	1799
Alloy-B10	11.45	−10.86	7.14	0.155	11.73	1.91	1815
Alloy-B15	11.88	−15.02	6.91	0.163	13.96	1.46	1848

**Table 4:** Formation criteria based on physical and chemical parameters using HEAPS program.

Nomenclature	MC1 (∆H_mix_-δ_r_)	MC2 (Ω-δ_r_)	MC7 (T/T_m_-δ_r_-∆H_mix_)	IMF1 (∆X^P^)
Alloy-B0	SS	SS	IM(0.5 < T/Tm < 0.9)	Uncertain
Alloy-B5	IM/BMG	IM	IM(0.5 < T/Tm < 0.9)	TCP phase
Alloy-B7	IM/BMG	IM	IM(0.5 < T/Tm < 0.9)	TCP phase
Alloy-B10	IM/BMG	IM	IM(0.5 < T/Tm < 0.9)	TCP phase
Alloy-B15	IM/BMG	IM	IM(0.5 < T/Tm < 0.9)	TCP phase

**Table 5:** Stacking Fault Energies (SFE) obtained with CALPHAD.

Alloys	$G^{\gamma }$ (J/mol)	$G^{\varepsilon }$ (J/mol)	$\Delta G^{\gamma \to \varepsilon }$ (J/mol)	a (Å)	ρ (mol/m^2^)	SFE (mJ/m^2^)
Alloy-B0	−7327.51	−7088.64	239	3.6	2.96 × 10^−5^	24.19
Alloy-B5	−15,300.30	−14,855.36	445	3.6	2.96 × 10^−5^	36.34
Alloy-B7	−17,181.93	−16,360.73	821	3.6	2.96 × 10^−5^	56.61
Alloy-B10	−17,059.02	−16,206.17	853	3.6	2.96 × 10^−5^	60.48
Alloy-B15	−16,824.66	−15,911.14	913	3.6	2.96 × 10^−5^	64.08

**Table 6:** Root Mean Square Deviation (RMSD) to compare boron-added alloys.

Phase	Alloys	RMSD All Atoms	RMSD All Cr	RMSD All Mn	RMSD All Fe	RMSD All Co
FCC	alloy-B5 vs. alloy-B7	2.824	4.758	2.705	2.163	3.505
HCP	alloy-B5 vs. alloy-B7	2.209	0.671	3.041	2.007	0.666
FCC	alloy-B10 vs. alloy-B15	2.247	0.546	2.205	1.726	4.039
HCP	alloy-B10 vs. alloy-B15	1.656	0.441	0.333	2.437	0.310

**Table 7:** Total energy for the alloys studied and magnetic corrections for FCC structures.

Alloys	E Total FCC (eV)	E Total HCP (eV)	E_AFM_ (eV)	E_FM_ (eV)	E_AFM/FM_ (eV)
Alloy-B0	−487.98	−502.23	−494.87	−495.91	−494.64
Alloy-B5	−500.22	−505.97	−506.97	−507.22	−506.85
Alloy-B7	−514.69	−521.81	−513.21	−513.84	−513.08
Alloy-B10	−506.49	−504.27	−506.76	−506.85	−506.53
Alloy-B15	−513.18	−511.34	−515.10	−515.23	−514.99

**Table 8:** Enthalpy of formation for the alloys (Fe_50_Mn_30_Cr_10_Co_10_)-xBx (x = 0, 5, 7, 10, and 15 at.%). Values are in kJ/mol.

Alloys	∆Hf (kJ/mol), FCC	∆Hf (kJ/mol), HCP
Alloy-B0	−14.09	−14.19
Alloy-B5	−14.08	−14.24
Alloy-B7	−14.44	−14.33
Alloy-B10	−14.26	−14.18
Alloy-B15	−14.39	−14.34

**Table 9:** Stacking Fault Energies (SFE) obtained with DFT.

Alloys	ΔE_HCP-FCC_ (eV)	SFE_ANNI_ (mJ/m^2^)	SFE_ANNI+M2B_ (mJ/m^2^)
Alloy-B0	6.66	20.74	-
Alloy-B5	5.75	18.34	-
Alloy-B7	7.11	23.00	-
Alloy-B10	2.22	6.18	52.55
Alloy-B15	1.84	5.07	51.44

## Data Availability

Not applicable.
